# Residual Quantitative Flow Ratio to Estimate Post‐Percutaneous Coronary Intervention Fractional Flow Reserve

**DOI:** 10.1155/2021/4339451

**Published:** 2021-08-31

**Authors:** Pepijn A. van Diemen, Ruben W. de Winter, Stefan P. Schumacher, Michiel J. Bom, Roel S. Driessen, Henk Everaars, Ruurt A. Jukema, Yvemarie B. Somsen, Lenka Popelkova, Peter M. van de Ven, Albert C. van Rossum, Tim P. van de Hoef, Stefan de Haan, Koen M. Marques, Jorrit S. Lemkes, Yolande Appelman, Alexander Nap, Niels J. Verouden, Maksymilian P. Opolski, Ibrahim Danad, Paul Knaapen

**Affiliations:** ^1^Department of Cardiology, Amsterdam UMC, Vrije Universiteit Amsterdam, Amsterdam, Netherlands; ^2^Department of Epidemiology and Biostatistics, Amsterdam UMC, Vrije Universiteit Amsterdam, Amsterdam, Netherlands; ^3^Department of Interventional Cardiology and Angiology, National Institute of Cardiology, Warsaw, Poland

## Abstract

**Objectives:**

Quantitative flow ratio (QFR) computes fractional flow reserve (FFR) based on invasive coronary angiography (ICA). Residual QFR estimates post‐percutaneous coronary intervention (PCI) FFR. This study sought to assess the relationship of residual QFR with post-PCI FFR.

**Methods:**

Residual QFR analysis, using pre-PCI ICA, was attempted in 159 vessels with post-PCI FFR. QFR lesion location was matched with the PCI location to simulate the performed intervention and allow computation of residual QFR. A post-PCI FFR < 0.90 was used to define a suboptimal PCI result.

**Results:**

Residual QFR computation was successful in 128 (81%) vessels. Median residual QFR was higher than post-PCI FFR (0.96 Q1–Q3: 0.91–0.99 vs. 0.91 Q1–Q3: 0.86–0.96, *p* < 0.001). A significant correlation and agreement were observed between residual QFR and post-PCI FFR (*R* = 0.56 and intraclass correlation coefficient = 0.47, *p* < 0.001 for both). Following PCI, an FFR < 0.90 was observed in 54 (42%) vessels. Specificity, positive predictive value, sensitivity, and negative predictive value of residual QFR for assessment of the PCI result were 96% (95% confidence interval (CI): 87–99%), 89% (95% CI: 72–96%), 44% (95% CI: 31–59%), and 70% (95% CI: 65–75%), respectively. Residual QFR had an accuracy of 74% (95% CI: 66–82%) and an area under the receiver operating characteristic curve of 0.79 (95% CI: 0.71–0.86).

**Conclusions:**

A significant correlation and agreement between residual QFR and post-PCI FFR were observed. Residual QFR ≥ 0.90 did not necessarily commensurate with a satisfactory PCI (post-PCI FFR ≥ 0.90). In contrast, residual QFR exhibited a high specificity for prediction of a suboptimal PCI result.

## 1. Introduction

The assessment of the functional severity of coronary lesions by fractional flow reserve (FFR) has shown to favorably guide revascularization therapy in terms of symptom alleviation and prognosis as compared to medical therapy and/or invasive angiography alone [1–4]. Although the use of FFR to establish the significance of coronary artery disease (CAD) is common practice, FFR is not routinely measured post‐intervention, while studies have demonstrated that a percutaneous coronary intervention (PCI) with a suboptimal functional outcome is associated with an increased risk of future adverse events and symptomatic burden [5–8]. Furthermore, FFR measurements post-PCI can help the interventionalist identify vessels that, e.g., due to the presence of stent-related issues and/or residual focal stenosis may benefit from additional stent optimization and/or stent placement [9]. Notwithstanding the aforementioned benefits of post-PCI FFR interrogation, the use of intracoronary wires is associated with a small risk of serious complications (not to mention prolonged procedural time and additional costs) and thus infrequently applied in clinical practice [5]. As an alternative to FFR, quantitative flow ratio (QFR) applies fluid dynamics to a 3D model of the coronaries derived from invasive coronary angiography (ICA) images to accurately compute pre-PCI FFR, obviating the need for intracoronary pressure wires and hyperemic agents [10–13]. QFR analysis furthermore allows for the computation of residual QFR, which is an estimation of post-PCI FFR if a selected lesion were to be treated. This study assessed the correlation between residual QFR and post-PCI FFR and the ability of residual QFR to predict a suboptimal PCI result defined by post-PCI FFR.

## 2. Methods

### 2.1. Study Population

This study retrospectively evaluated patients from the On-Site Computed Tomography Versus Angiography Alone to Guide Coronary Stent Implantation trial (AR-PCI trial, NCT03531424), the Prospective Comparison of Cardiac PET/CT, SPECT/CT Perfusion Imaging, and CT Coronary Angiography With Invasive Coronary Angiography trial (PACIFIC trial, NCT01521468), and the Registry of Patients That Have Undergone ICA or PCI (ICA-PI registry, NCT04815928) for inclusion [14, 15]. The AR-PCI trial randomized 60 patients with stable CAD and a clinical indication for PCI to coronary computed tomography angiography- (CCTA-) guided PCI or angiography-guided PCI [15]. The PACIFIC trial included 208 patients with suspected stable CAD who underwent single-photon emission computed tomography, positron emission tomography, and CCTA prior to ICA with interrogation of all major coronary arteries by FFR [14]. The ICA-PI registry is an ongoing registry that includes patients that have undergone a clinically indicated ICA and/or PCI at the Amsterdam UMC: VUmc, Amsterdam, The Netherlands. The inclusion and exclusion criteria of the AR-PCI trial and PACIFIC trial are presented in Supplemental Information 1 and 2. For the present study, patients were eligible for inclusion if FFR measurements were obtained after an angiographically successful PCI as determined by the operator. Patients were excluded if complications occurred during PCI, if additional interventions were performed following the post-PCI FFR measurement, if patient or lesion characteristics prohibited QFR analysis, or if ICA images were unsuitable for QFR analysis (Supplemental Information 3). The study was approved by the institutional ethics committee and complied with the Declaration of Helsinki. All patients provided written informed consent.

### 2.2. Percutaneous Coronary Intervention and Fractional Flow Reserve

Invasive angiography was performed using a standard protocol on a monoplane cardiovascular X-ray system (Allura Xper FD 10/10, Philips Healthcare, Best, the Netherlands) visualizing the coronary artery of interest in at least 2 directions. Prior to contrast injection, 0.2 ml nitroglycerine was injected to induce epicardial coronary vasodilation. Angiography images were not systematically obtained using a dedicated QFR acquisition protocol. After acquisition of ICA images, FFR was measured using a pressure sensor-tipped guidewire (Volcano Corporation, San Diego, United States of America). Maximal hyperemia was induced by intracoronary (150 *µ*g) or intravenous (140 *µ*g/kg/min) administration of adenosine. FFR was calculated as the ratio between the mean distal coronary pressure and mean aortic pressure. A lesion with an FFR ≤ 0.80 was deemed hemodynamically significant [3, 4]. An FFR of 0.50 was assigned to subtotal lesions in which wire passage was not deemed possible [16]. In the ICA-PI registry and PACIFIC trial, the procedural plan (e.g., stent length/diameter and pre/post‐dilation) of the intervention was left at the discretion of the operator. In the AR-PCI trial, the procedural plan of the PCI was based on angiography and left at the discretion of the operator or predetermined based on CCTA findings, depending on the randomization assignment [15]. Post-PCI FFR was performed similarly as pre-PCI FFR. A post-PCI FFR < 0.90 was used to define a suboptimal PCI result [8].

### 2.3. Quantitative Flow Ratio Analysis

**Figure 1:**
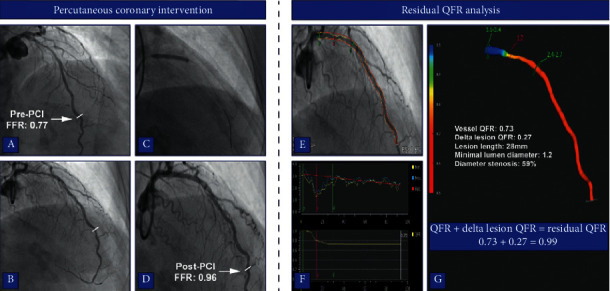
Case example of residual QFR analysis. (a-b) Two end-diastolic frames of ICA in a patient with a proximal LAD lesion (FFR: 0.77). The stenosis was angiographically and functionally (post-PCI FFR: 0.96) successfully revascularized (c-d). During the QFR analysis, vessel contours were automatically detected on the two end-diastolic frames and manually corrected if needed (e). A 3D model of the coronary was reconstructed based on the vessels contours and lesions are presented as a drop in vessel diameter and QFR (g-f). The proximal and distal markers of the lesion in the QFR analysis were matched with the treated location during PCI to allow virtual removal of the lesion (c, f-g). Residual QFR was calculated by adding the delta QFR of the lesion to the vessel QFR (g). FFR: fractional flow reserve, ICA: invasive coronary angiography, LAD: left anterior descending artery, PCI: percutaneous coronary intervention, and QFR: quantitative flow ratio.

Computation of QFR was retrospectively performed blinded to FFR measurements using QAngio XA 3D/QFR V2.0 solution software package (Medis Medical Imaging, Leiden, the Netherlands). Two pre-PCI end-diastolic frames from the coronary of interest that were at least 25° apart were used to reconstruct a 3D-model of the vessel. QFR analyses were performed up to the location of the post-PCI pressure wire location. The reference diameter of the vessel was constructed by marking healthy segments within the coronary artery of interest. An estimation of contrast velocity was obtained by contrast frame counting of the analyzed artery. The estimated contrast velocity was converted into a virtual hyperemic flow velocity that allowed for the computation of the pressure drop and QFR along the analyzed coronary. For vessels (*N* = 6) in which contrast counting could not be accurately performed, a fixed hyperemic flow velocity was used to compute the pressure drop and QFR. Proximal and distal markers of the lesion were matched with the proximal and distal locations of the treated area during the performed PCI. Residual QFR was calculated by adding the delta QFR of the analyzed lesion to the vessel QFR (Figure 1). Similar to FFR, a vessel with a pre-PCI QFR ≤ 0.80 was indicative of significant CAD and a residual QFR < 0.90 was used as a threshold to define a predicted suboptimal PCI result [8, 17].

### 2.4. Statistical Analysis

Statistical analyses were performed using SPSS software package version 26 (IBM SPSS Statistics, Armonk, United States of America) and MedCalc (version 12.7.8.0, MedCalc Software, Oostende, Belgium). Continuous variables with a normal distribution are presented as mean with standard deviation, whereas continuous variables with a nonnormal distribution are presented as median with Q1–Q3. Categorical variables are displayed as numbers with percentages. The correlation between QFR and pre-PCI FFR was quantified using Pearson's correlation coefficient and the correlation between residual QFR and post-PCI FFR with Spearman's correlation coefficient. Agreement of QFR and pre-PCI FFR and of residual QFR and post-PCI FFR was assessed using the Bland–Altman analysis and quantified using an intraclass correlation coefficient which was calculated using a two-way mixed model for the absolute agreement for single measures. To account for clustering of multiple vessel measurements per patient, differences between QFR and pre-PCI FFR and residual QFR and post-PCI FFR were compared using a mixed linear model with a fixed effect for the technique and random effects for patient and vessels nested within a patient. The diagnostic performance of residual QFR to assess a post-PCI FFR < 0.90 was calculated as sensitivity, specificity, positive predictive value (PPV), negative predictive value (NPV), accuracy, and area under the receiver operating characteristic (ROC) curve with corresponding 95% confidence interval. A two-sided *p* value <0.05 was considered statistically significant. Data supporting the results of the present study can be made available upon reasonable request.

## 3. Results

### 3.1. Study Population

**Figure 2:**
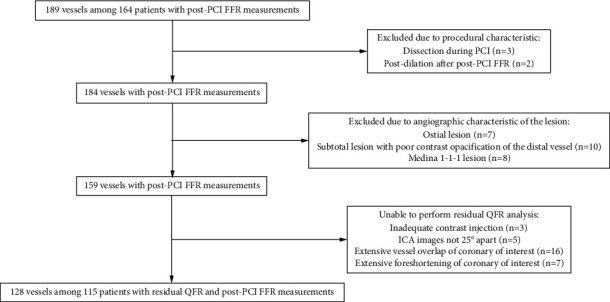
Flowchart of patients and vessels that were included and excluded. AUC, area under receiver operating characteristic curve; FFR, fractional flow reserve; PCI, percutaneous coronary intervention; QFR, quantitative flow ratio; ICA, invasive coronary angiography.

A total of 189 vessels with post-PCI FFR measurements among 164 patients were evaluated for inclusion. Five vessels were excluded due to procedural characteristics (complication during PCI (*N* = 3) and post‐dilation after post-PCI FFR (*N* = 2)), and an additional 25 were excluded as result of the angiographic characteristics of the lesion (Figure 2). Residual QFR analysis was attempted in the remaining 159 vessels and was unsuccessful in 31 (19%) vessels due to poor contrast opacification (2%), ICA images not 25° apart (3%), extensive vessel overlap (10%), or extensive foreshortening (4%). As such, the final study population consisted of 115 patients (77% male, age: 64.9 ± 9.9 years) with 128 vessels in which post-PCI FFR measurements and residual QFR analyses were performed. Nearly half (*N* = 55, 48%) of the patients had a previous PCI and 25% (*N* = 29) a prior myocardial infarction. Ninety-nine percent (*N* = 114) of the study population presented with stable CAD, whereas 1% (*N* = 1) had a non-ST segment elevation myocardial infarction (Table 1).

### 3.2. Angiography and Procedural Characteristics

Angiographic and procedural characteristics are presented in Tables 2 and 3. A total of 134 lesions with an average diameter stenosis of 64 ± 12% were treated among the 128 vessels. Fifty-four percent (*N* = 72) of the lesions were located in the LAD territory, followed by the RCA (*N* = 37, 28%) and lastly the Cx territory (*N* = 25, 19%). Predilation was applied in 64 (50%) procedures. In 90 (70%) vessels, a single stent was implanted, whereas in 31 (24%) and 5 (4%) vessels, 2 and 3 stents were necessary, respectively. Two vessels were treated with drug-coated balloons only. Following stent implantation, post‐dilation was used in 94 (73%) vessels.

### 3.3. Correlation and Agreement of QFR Indices and FFR

**Figure 3:**
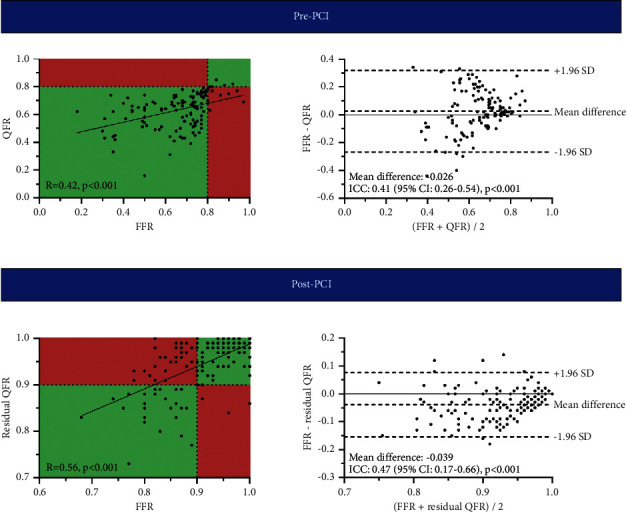
Correlation and agreement between QFR and pre-PCI FFR and residual QFR and post-PCI FFR. Scatterplots with corresponding correlation coefficients and Bland–Altman plots with ICCs demonstrating the correlation and level of agreement between QFR and pre-PCI FFR and residual QFR and post-PCI FFR. FFR, fractional flow reserve; PCI, percutaneous coronary intervention; QFR, quantitative flow ratio; CI, confidence interval; ICC, intraclass correlation coefficient; SD, standard deviation.

Table 4 presents the physiological vessel characteristics of the studied population. Prior to PCI, 117 (91%) vessels had an FFR ≤ 0.80, whereas 125 (98%) vessels had a QFR ≤ 0.80. QFR was lower than pre-PCI FFR (0.63 ± 0.12 vs. 0.66 ± 0.15, *p*=0.049). QFR and pre-PCI FFR correlated and were in agreement with one another (*R* = 0.42, *p* < 0.001, and ICC = 0.41, *p* < 0.001, Figure 3). The diagnostic performance of QFR to predict significant CAD defined by pre-PCI FFR is presented in Table 5. Residual QFR was higher compared to post-PCI FFR (0.96 Q1–Q3: 0.91–0.99 vs. 0.91 Q1–Q3: 0.86–0.96, *p* < 0.001). Residual QFR correlated and was in agreement with post-PCI FFR (*R* = 0.56, *p* < 0.001, and ICC = 0.47, *p* < 0.001).

### 3.4. Residual QFR as a Predictor of a Suboptimal PCI Result

**Figure 4:**
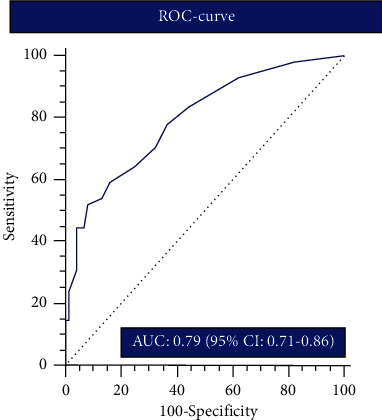
Diagnostic performance of residual QFR for detecting a suboptimal PCI result. ROC curve with AUC demonstrating the performance of residual QFR to predict a suboptimal PCI result defined by post-PCI FFR < 0.90. AUC, area under receiver operating characteristics curve; FFR, fractional flow reserve; PCI, percutaneous coronary intervention; QFR, quantitative flow ratio; CI, confidence interval; ROC, receiver operator characteristics.

**Figure 5:**
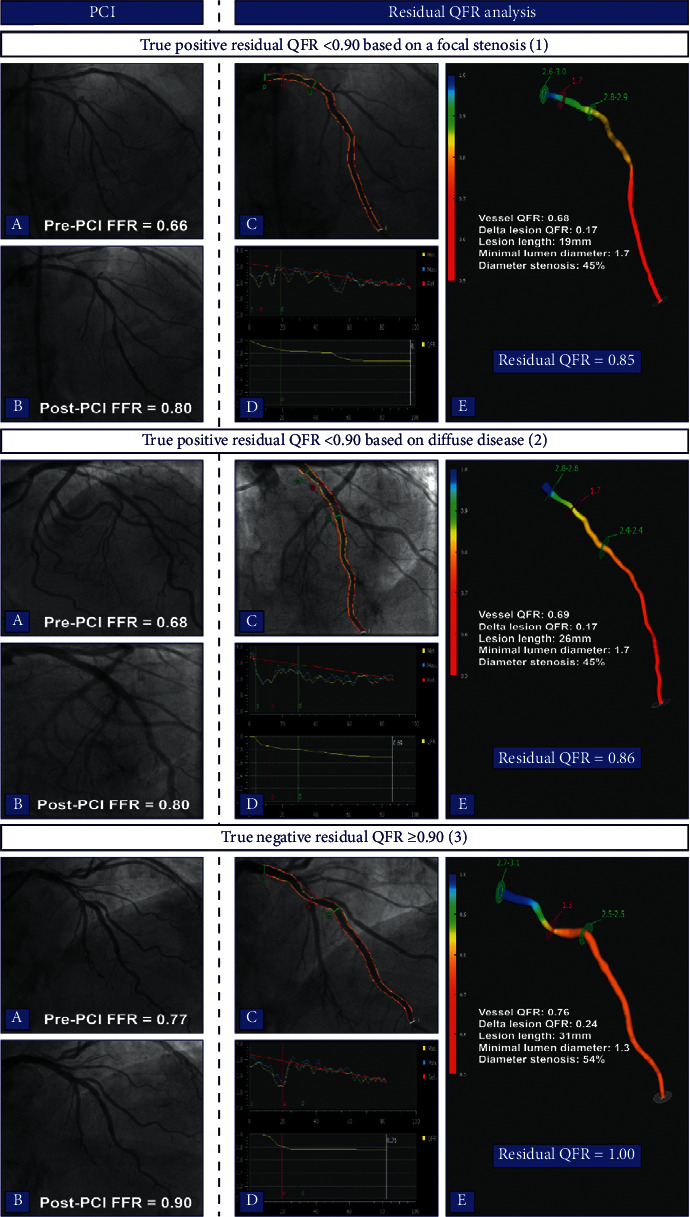
Case examples of true positive and true negative residual QFR results. Case 1 demonstrates a case of a successfully treated proximal LAD lesion with a suboptimal post-PCI FFR (1a-b). Residual QFR estimated a post-PCI FFR of 0.85 (1c–e). The suboptimal result was caused by a pressure drop due to an additional lesion in the mid-LAD (1d). In case 2, residual QFR was 0.86 and FFR after treating the proximal LAD lesion was 0.80 (2a–e). QFR analyses revealed diffuse disease without a focal pressure drop to be the potential cause of the suboptimal result (2D). The last case demonstrates a case of a true negative result; the lesion in the proximal LAD was successfully treated, and there were no additional lesions or diffuse disease which led to a concordant residual QFR and post-PCI FFR ≥ 0.90 (3a–e). FFR, fractional flow reserve; PCI, percutaneous coronary intervention; QFR, quantitative flow ratio; LAD, left anterior descending artery.

**Figure 6:**
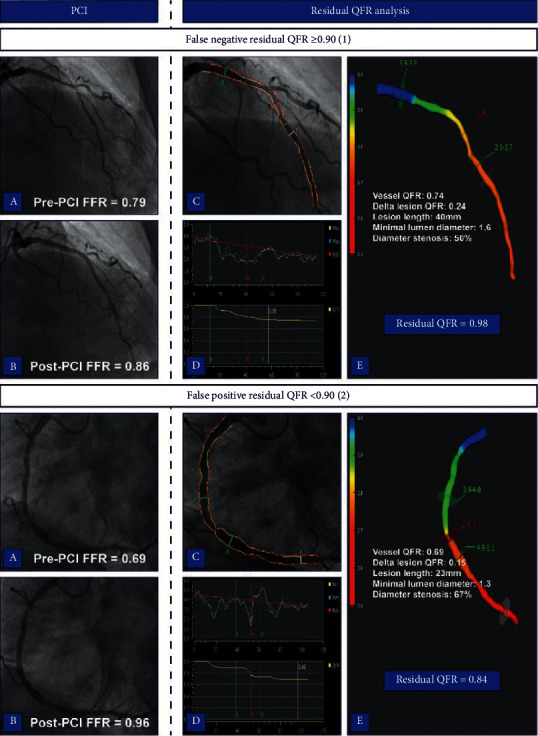
Case examples of false negative and false positive residual QFR results. Case 1 displays a false negative residual QFR result. Post-PCI FFR remained suboptimal after a successful intervention in proximal to mid LAD (1a-b). Residual QFR estimated a post-PCI FFR of 0.98, and there was no significant pressure drop observed beside that of the treated lesion (1c–e). Contrary, case 2 shows a satisfactory PCI result of the RCA (2a-b). However, residual QFR predicted a suboptimal PCI result based on the presence of additional stenosis that proved not to be functionally relevant (2c–e). FFR, fractional flow reserve; PCI, percutaneous coronary intervention; QFR, quantitative flow ratio; LAD, left anterior descending artery; RCA, right coronary artery.

A suboptimal PCI result defined as a post-PCI FFR < 0.90 was observed in 54 (42%) vessels, whereas residual QFR < 0.90 was seen in 27 (21%) vessels. Sensitivity, specificity, PPV, and NPV of residual QFR to predict post-PCI FFR < 0.90 were 44%, 96%, 89%, and 70%, respectively (Table 5). Overall, residual QFR had an accuracy of 74% and AUC of 0.79 for determining a suboptimal PCI result (Figure 4). Figures 5 and 6 present case examples of true positive, true negative, false positive, and false negative residual QFR results.

## 4. Discussion

The present study assessed the ability of angiography-based residual QFR to estimate post-PCI FFR and its value to predict a suboptimal PCI result defined by a post-PCI FFR < 0.90. Among 128 vessels, a significant correlation and agreement between residual QFR and post-PCI FFR was observed. The performance of residual QFR to define a suboptimal PCI result was characterized by a high specificity and PPV, whereas overall accuracy was hampered by a low sensitivity and moderate NPV. These exploratory observations may indicate that QFR analyses with residual QFR calculation might aid the interventionalist in determining a treatment strategy as it is able to detect residual focal and/or diffuse disease that may lead to an unsatisfactory post-PCI FFR.

### 4.1. QFR and Pre-PCI FFR

The diagnostic performance of QFR for assessment of significant CAD as defined by FFR has been validated in four prospective trials that collectively included 969 vessels [17]. In a meta-analysis of the diagnostic trials, QFR exhibited a sensitivity and specificity of 85% and 88%, respectively [17]. Furthermore, a good correlation between QFR and FFR was observed (*R* = 0.80) [17]. Importantly, the diagnostic performance observed in the present study is not representative of that of QFR usage in clinical practice, as all patients underwent PCI and had for the vast majority FFR-defined significant CAD (91%). Regarding the correlation of QFR and FFR, we observe a lower correlation coefficient as compared to the meta-analysis of the diagnostic performance studies [17]. This observation may be driven by several factors. First, the present study included 128 vessels as compared to the 969 vessels in the meta-analysis, whereby, in general, a larger study population will lead to a higher correlation coefficient. Second, lesions with an FFR and QFR > 0.80 that populate the upper right quadrant of a correlation plot are virtually absent in our study. Inclusion of these lesions would, predictably, alter and improve the correlation coefficient. Last, the diameter stenosis percentage (DS%) in our study is higher than in the meta-analysis. Increasing DS% is a significant predictor of a QFR at least 0.10 points lower than FFR, as such the difference in DS% may have lead to a lower correlation coefficient in our study [17].

### 4.2. Residual QFR and Post-PCI FFR

Data regarding the comparsion of residual QFR with post-PCI FFR is scarce. The association of residual QFR with post-PCI FFR has been assessed among 84 vessels in a substudy of the Does Optical Coherence Tomography Optimize Results of Stenting (DOCTORS) Study [18] Rubimbura et al. observed a significant correlation (*R* = 0.68) between residual QFR and post-PCI FFR [18]. In the present study, residual QFR and post-PCI FFR correlated to a lesser extent (*R* = 0.56), possibly due to differences in the included study population. In contrast to our study, the DOCTORS substudy excluded vessels with in-stent restenosis, severely calcified or tortuous vessels, one or more other lesions considered significant on angiography, or nonsignificant diffuse disease of the target-vessel. These characteristics influence the complexity of the PCI procedure and consequently increase the chance of a suboptimal result [19–21]. Of note, residual QFR calculation assumes that stent placement and deployment of the analyzed lesion are optimal. For example, PCI of a complex calcified lesion may yield a suboptimal PCI outcome and as such can result in discordancy between post-PCI FFR and residual QFR based on the assumption of an optimal PCI result by the latter [19].

### 4.3. Post-PCI FFR as a Determinant of Outcome and the Ability of Residual QFR to Predict a Suboptimal PCI Result

The increase in FFR that is achieved by revascularization is directly associated with restoration of myocardial perfusion as measured by [^15^O]H_2_O positron emission tomography and is related to the extent of symptomatic relieve [7, 22]. Nevertheless, in some patients, an angiographically successful PCI does not leads to an FFR above the “ischemic” threshold (FFR > 0.80), which may partly explain that 20–30% of patients report recurrent angina 1 year post-PCI [1, 5, 9, 20, 23, 24]. In the present study, 10% of the revascularized vessels had a post-PCI FFR ≤ 0.80 and 42% had a suboptimal PCI result (FFR < 0.90). These rates are in line with the study of Diletti et al., in which 7.7% and 37.8% of the vessels had a post-PCI FFR ≤ 0.80 and < 0.90, respectively [23]. Interestingly, vessels with an unsatisfactory post-PCI FFR might be amenable to additional interventions (e.g., post‐dilation or additional stent placement) that can augment FFR [9, 19]. Furthermore, post-PCI FFR has been established as an important prognostic marker by numerous studies [1, 5, 6, 25–28]. Clinical applicability of post-PCI FFR is, however, hampered by the lack of a universally accepted threshold to delineate a functionally suboptimal PCI and absence of randomized trials studying treatment strategy based on post-PCI FFR [5]. Rimac et al. have attempted to define a post-PCI threshold in a meta-analysis of 59 studies and demonstrated that a post-PCI FFR < 0.90 was associated with an increased risk of repeat revascularization and major adverse cardiac events (MACE) [8]. Recently, Diletti et al. assessed this prognostic threshold among 959 patients with post-PCI FFR and showed that the cutoff was associated with target-vessel revascularization [23]. In line with these publications, a post-PCI FFR < 0.90 was used to define a suboptimal PCI result in our study. In general, a suboptimal PCI result is caused by stent-related issues or residual atherosclerotic disease outside the stented area [5]. Residual QFR is able to detect residual focal and/or diffuse disease that may lead to a suboptimal post-PCI FFR with satisfactory accuracy, as seen by the high PPV (Figure 5). Besides residual disease, stent-related issues are an important contributor to an unsatisfactory post-PCI FFR [5, 19]. In the study of Wolfrum et al., 21 (60%) vessels had a post-PCI FFR < 0.90 after an angiographically successful PCI [19]. Thirteen of the 21 (62%) vessels had a suboptimal stent result as defined by optical coherence tomography criteria, and subsequent stent optimization resulted in a significant increase in mean FFR from 0.80 to 0.88 [19]. Residual QFR analysis assumes optimal stent placement and deployment, as such stent-related issues (e.g., malapposition), are not taken into account when the post-PCI FFR is predicted. Therefore a suboptimal PCI result due to stent-related issues is not detected by residual QFR analyses which lowers its sensitivity and NPV.

### 4.4. Limitations

Although the present study is the largest to date assessing the relationship between residual QFR and post-PCI FFR, the total number of vessels is relatively limited and our results should be validated in larger cohorts. The present study lacks systematic intracoronary imaging to demonstrate the underlying causes of a suboptimal post-PCI FFR despite an angiographically successful intervention. Furthermore, in 19% of the vessels, QFR analyses could not be performed due to inadequate ICA images. Future prospective trials should utilize optimized QFR ICA acquisition protocols in order to minimize the rejection rate. Last, we manually designated the proximal and distal part of the analyzed lesion to match the treated location as accurately as possible; this might, however, have led to small inaccuracies that could have influenced the correlation and agreement results of residual QFR and post-PCI FFR.

## 5. Conclusions

Residual QFR < 0.90 is a good indicator of a suboptimal PCI result as defined by a post-PCI FFR < 0.90. In contrast, residual QFR ≥ 0.90 did not necessarily commensurate with a post-PCI FFR ≥ 0.90. These exploratory observations indicate that the high PPV of residual QFR is presumably driven by its ability to detect residual focal and/or diffuse disease outside the stented area. Therefore, QFR analyses might be used to assist the interventionalist in determining to most appropriate treatment strategy.

## Figures and Tables

**Table 1:** Patient characteristics.

Patient characteristics	*N* = 115
Male gender	89 (77%)
Age in years	64.9 ± 9.9
Body mass index (kg/m^2^)	26.7 ± 3.8

Cardiovascular risk factors	
Diabetes mellitus	18 (16%)
Hypertension	61 (53%)
Hypercholesterolemia	67 (58%)
Tobacco use	11 (10%)
History of tobacco use	58 (50%)
Family history of coronary artery disease	49 (44%)

Medication	
Acetylic acid	101 (88%)
P2Y12 inhibitor	36 (31%)
Beta-blockers	64 (56%)
Calcium-channel blockers	47 (41%)
Long-acting nitrates	23 (20%)
Statins	93 (81%)
Angiotensin-converting enzyme inhibitors	27 (24%)
Angiotensin receptor blockers	17 (15%)

Cardiac history	
Prior percutaneous coronary intervention	55 (48%)
Prior myocardial infarction	29 (25%)

Symptoms	
Typical angina pectoris	57 (50%)
Atypical angina pectoris	24 (21%)
Nonspecific chest pain	8 (7%)
Dyspnea	16 (14%)
Asymptomatic	10 (9%)

Indication for percutaneous coronary intervention	
Stable coronary artery disease	114 (99%)
Non-ST segment elevation myocardial infarction	1 (1%)

Values are presented as *N* (%) or mean ± SD.

**Table 2:** Angiographic characteristics.

Lesion location	134 lesions
Right coronary artery territory	
Right coronary artery	33 (25%)
Ramus descending posterior	3 (2%)
Right posterolateral	1 (1%)

Left anterior descending artery territory	
Left anterior descending artery	70 (52%)
Diagonal	2 (1%)

Circumflex artery territory	
Circumflex artery	14 (10%)
Obtuse marginal	8 (6%)
Anterolateral	3 (2%)

3D quantitative coronary angiography parameters	
Diameter stenosis	64 ± 12%
Area stenosis	81 ± 10%
Minimal lumen diameter (mm)	0.98 ± 0.36

Values are presented as *N* (%) or mean ± SD.

**Table 3:** Procedural characteristics.

Procedural characteristics	128 vessels
Lesions treated	
1	122 (95%)
2	6 (5%)

Number of stents implanted	
0^a^	2 (2%)
1	90 (70%)
2	31 (24%)
3	5 (4%)

Stent type^b^	
Drug-eluting stent	121 (95%)
Bare metal stent	2 (2%)
Bioresorbable stent	2 (2%)
Drug-coated balloon	2 (2%)

Stent size	
Total length implanted stents (mm)^c^	30 (20–45.75)
Nominal stent diameter (mm)^d^	3.25 ± 0.43
Stent diameter after implantation (mm)^e^	3.60 ± 0.53

Predilation and post‐dilation	
Predilation	64 (50%)
Post‐dilation	94 (73%)

Values are presented as *N* (%), mean ± SD, or median (Q1–Q3). ^a^Two vessels were only treated with drug-coated balloons. ^b^Not documented for one vessel. ^c^Not documented for 4 vessels. ^d^Not documented for 5 vessels. ^e^Not documented for 3 vessels.

**Table 4:** Physiological characteristics.

Physiological parameters	128 vessels
Pre-PCI	
QFR	0.63 ± 0.12
QFR ≤ 0.80	125 (98%)
FFR	0.66 ± 0.15
FFR ≤ 0.80	117 (91%)

Post-PCI	
Residual QFR	0.96 (0.91–0.99)
Residual QFR ≤ 0.80	4 (3%)
Residual QFR < 0.90	27 (21%)
FFR	0.91 (0.86–0.96)
FFR ≤ 0.80	13 (10%)
FFR < 0.90	54 (42%)

Values are presented as mean ± SD, number with %, or as median (Q1–Q3). FFR, fractional flow reserve; PCI, percutaneous coronary intervention; QFR, quantitative flow ratio.

**Table 5:** Diagnostic performance of QFR and residual QFR to assess significant CAD (pre-PCI FFR ≤ 0.80) and a suboptimal PCI outcome (post-PCI FFR < 0.90), respectively.

Diagnostic performance	128 vessels
QFR to detect pre-PCI FFR ≤ 0.80	
Sensitivity	100% (97–100%)
Specificity	27% (6–61%)
Negative predictive value	100% (not applicable)
Positive predictive value	94% (91–95%)
Accuracy	94% (88–97%)

Residual QFR to detect post-PCI FFR < 0.90	
Sensitivity	44% (31–59%)
Specificity	96% (87–99%)
Negative predictive value	70% (65–75%)
Positive predictive value	89% (72–96%)
Accuracy	74% (66–82%)

Values are presented as % (95% confidence interval). FFR, fractional flow reserve; PCI, percutaneous coronary intervention; QFR, quantitative flow ratio.

## Data Availability

The data used to support the findings of this study are available in the Amsterdam UMC, VUmc, and are available from the corresponding author upon reasonable request.

## Abbreviations

FFR: Fractional flow reserve
PCI: Percutaneous coronary intervention
QFR: Quantitative flow ratio
ICA: Invasive coronary angiography
CAD: Coronary artery disease
CCTA: Coronary computed tomography angiography
IQR: Interquartile range
PPV: Positive predictive value
NPV: Negative predictive value.

## Supplementary Materials

Supplementary Materials Supplemental Information 1 and 2 provide detailed inclusion and exclusion criteria of the On-Site Computed Tomography Versus Angiography Alone to Guide Coronary Stent Implantation: A Prospective Randomized Study (AR-PCI trial) and the Prospective Comparison of Coronary CT Angiography, SPECT, PET, and Hybrid Imaging for Diagnosis of Ischemic Heart Disease Determined by Fractional Flow Reserve Study (PACIFIC trial) from which patients were evaluated for inclusion in the present study. Supplemental information 3 provides a detailed list of patient, lesion, and invasive coronary angiography characteristics that prohibit QFR analysis.
