# Supplementary material for: Residual Quantitative Flow Ratio to Estimate Post‐Percutaneous Coronary Intervention Fractional Flow Reserve
Source: J Interv Cardiol. 2021 Aug 31;2021:4339451. doi: 10.1155/2021/4339451 (PMC8426071; doi:10.1155/2021/4339451)
Supplement: Supplementary Materials — Supplemental Information 1 and 2 provide detailed inclusion and exclusion criteria of the On-Site Computed Tomography Versus Angiography Alone to Guide Coronary Stent Implantation: A Prospective Randomized Study (AR-PCI trial) and the Prospective Comparison of Coronary CT Angiography, SPECT, PET, and Hybrid Imaging for Diagnosis of Ischemic Heart Disease Determined by Fractional Flow Reserve Study (PACIFIC trial) from which patients were evaluated for inclusion in the present study. Supplemental information 3 provides a detailed list of patient, lesion, and invasive coronary angiography characteristics that prohibit QFR analysis. [file 4339451.f1.pdf]

Supplemental information 1. In- and exclusion criteria of the On-Site Computed Tomography Versus Angiography Alone to Guide Coronary Stent Implantation: A Prospective Randomized Study (AR-PCI trial) (1).

Eligible patients had at least 1 target lesion located in a native coronary artery with a visually estimated reference vessel diameter of at least 2.25 mm without any upper limits.

Exclusion criteria entailed:

-Insufficient quality of coronary computed tomography angiography.

-Renal failure (estimated glomerular filtration rate <30 mL/min).

-Known allergy to contrast medium.

-Percutaneous coronary intervention (PCI) of the left main coronary.

-PCI of bifurcation lesions in which strategies other than a single crossover stent technique were anticipated.

-PCI of in-stent restenosis.

-PCI of bypass grafts.

-PCI of chronically occluded vessels.

Supplemental information 2. In- and exclusion criteria of The Prospective Comparison of Coronary CT Angiography, SPECT, PET, and Hybrid Imaging for Diagnosis of Ischemic Heart Disease Determined by Fractional Flow Reserve (PACIFIC-trial) (2).

Inclusion criteria:

-First presentation to cardiologist with suspected coronary artery disease (CAD).

-No documented prior history of CAD.

-Intermediate pre-test likelihood of CAD as defined by Diamond and Forrester criteria.

-Referred for a clinically indicated invasive coronary angiography.

-Age >40 years.

Exclusion criteria:

-History of severe chronic obstructive pulmonary disease or chronic asthma.

-Pregnancy.

-Renal failure (estimated glomerular filtration rate <45 mL/min).

-Use of sildenafil or dipyridamole that cannot be terminated.

-Contra-indication for β-blockers.

-Known allergy for iodinated contrast.

-Concurrent or prior (within last 30 days) participation in other studies using investigational drugs.

-Claustrophobia.

-Significant co-morbidities.

-Atrial fibrillation, second- or third-degree atrioventricular block.

-Tachycardia.

-Suspected acute myocardial infarction.

-Prior percutaneous coronary intervention or coronary artery bypass grafting.

-Heart failure.

-Left ventricular ejection fraction <50%.

-Documented cardiomyopathy.

-Previous radiation exposure during the diagnostic work-up.

-Subject intended for short-term medical treatment or invasive coronary intervention.

Supplemental information 3. Patient, lesion, and invasive coronary angiography characteristics prohibiting QFR analysis.

Patient characteristics:

-Tachycardia with a frequency above 100 beats per minute.

-Systolic aortic resting blood pressure below 75mm Hg.

-Atrial fibrillation during invasive coronary angiography.

Lesion characteristics:

-Culprit lesions of patients with an acute coronary syndrome.

-Bifurcation lesions with a Medina 1-1-1 classification.

-Aorto-ostial artery stenosis or ostial right coronary artery stenosis.

-Distal left main lesions in combination with a proximal circumflex lesion.

-Vessels with retrograde filling.

-Bypass grafts.

-Grafted coronary arteries.

-Myocardial bridging.

Invasive coronary angiography characteristics:

-Inadequate contrast injection leading to poor contrast opacification of the vessel.

-Too much overlap of other vessels with the lesion or areas just around the lesion in the target vessel in one or both of the angiographic acquisitions.

-Too much foreshortening of the target coronary artery in one or both angiographic acquisitions.

Reference list

1. Opolski MP, Schumacher SP, Verouden NJW, van Diemen PA, Borucki BA, Sprengers R, et al. On-Site Computed Tomography Versus Angiography Alone to Guide Coronary Stent Implantation: A Prospective Randomized Study. J Invasive Cardiol. 2020;32(11):E268-e76.

2. Danad I, Raijmakers PG, Driessen RS, Leipsic J, Raju R, Naoum C, et al. Comparison of Coronary CT Angiography, SPECT, PET, and Hybrid Imaging for Diagnosis of Ischemic Heart Disease Determined by Fractional Flow Reserve. Jama Cardiology. 2017;2(10):1100-7.
